# Progestogen supplementation to prevent miscarriage: Evidence and controversies

**DOI:** 10.1002/ijgo.70793

**Published:** 2026-01-07

**Authors:** Gabriele Saccone, Vincenzo Berghella, Moti Gulersen, Attilio Di Spiezio Sardo

**Affiliations:** ^1^ Department of Neuroscience, Reproductive Sciences and Dentistry, School of Medicine University of Naples Federico II Naples Italy; ^2^ Division of Maternal Fetal Medicine Thomas Jefferson University Philadelphia Pennsylvania USA; ^3^ Department of Public Health, School of Medicine University of Naples Federico II Naples Italy

**Keywords:** abortion, miscarriage, progesterone

## Abstract

Progesterone plays a key role in the establishment and maintenance of pregnancy and its deficiency has been associated with early pregnancy loss. Progestogen supplementation has therefore been widely investigated as a preventive strategy against miscarriage, particularly in women with recurrent pregnancy loss or threatened miscarriage or after assisted reproductive technologies. However, the overall evidence remains inconclusive. Meta‐analyses of randomized trials, including the large PROMISE and PRISM studies, have not demonstrated a significant overall benefit in live birth rates, although a possible advantage was observed in women with both early bleeding and prior miscarriages. The biological plausibility of supplementation lies in the luteal–placental shift, when the placenta assumes progesterone production after the first trimester. Yet, low progesterone levels in early pregnancy might often represent a marker of a non‐viable gestation rather than the underlying cause of miscarriage. The optimal route and duration of treatment remain uncertain. Vaginal and subcutaneous progesterone offer comparable efficacy to intramuscular formulations, with better tolerability and compliance. Current evidence does not support universal progesterone supplementation in early pregnancy, but individualized treatment might be considered for women at increased risk, based on clinical history. Future research should focus on identifying reliable markers of luteal deficiency, refining patient selection and comparing different administration routes and serum thresholds.

## 1 INTRODUCTION

Progestogens are a class of natural or synthetic steroid hormones that activate the progesterone receptors.^1^ The term progestogens includes both progestins and natural progesterone. Progestins are synthetic progestogens and are used in medicine.^2^ Major examples of progestins include 17‐hydroxyprogesterone caproate, dydrogesterone, or 19‐nortestosterone derivative norethisterone. Natural progesterone is the main and most important progestogen in the body and has numerous physiological effects that are amplified in the presence of estrogens.^3^ One example of this is in breast tissue, where estrogens allow progesterone to mediate lobuloalveolar development.^4^ In obstetrics, progestogens are commonly used to treat or prevent different conditions, including first trimester abortion^5^ and preterm labor.^6^, ^7^, ^8^, ^9^, ^10^, ^11^, ^12^, ^13^, ^14^, ^15^

### 1.1 Biology and rationale for progesterone use in early pregnancy

Progesterone plays a central role in the establishment and maintenance of early pregnancy by promoting endometrial receptivity, supporting implantation and sustaining decidualization.^2^ In the first weeks of gestation, progesterone is produced predominantly by the corpus luteum, until the luteal–placental shift occurs at around 8 weeks, when the placenta becomes the primary source.^1^ This transition is crucial for the continuation of pregnancy, and inadequate progesterone production during this period has been hypothesized to compromise early placentation in a subset of women.^15^ Beyond its endocrine effects, progesterone has important immunomodulatory functions that facilitate maternal immune tolerance to the semi‐allogeneic fetus by modulating local immune cell activity and reducing inflammatory responses within the reproductive tract.^16^, ^17^, ^18^ The route of administration might influence these immunological effects, as vaginal progesterone can achieve high local concentrations through the vagina‐to‐uterus transport, whereas intramuscular formulations produce higher systemic levels.^19^

## 2 PROGESTOGENS IN FIRST TRIMESTER ABORTION

Miscarriage is defined as pregnancy loss occurring before 20 weeks of gestation.^16^ Up to 15% of all pregnancies end in an early spontaneous abortion.^17^ Known risk factors for first trimester abortion include advancing maternal age, smoking, ethnicity, overweight, or obesity.^18^ Progesterone is essential for establishment of a pregnancy. Withdrawal of progesterone in early pregnancy typically results in an abortion, and antiprogesterone drugs, such as mifepristone, are used for induced abortion.^16^, ^17^, ^18^ The central role of progesterone in maintenance of pregnancy led to the hypothesis that progesterone deficiency in the first trimester of pregnancy can cause abortion. Therefore, several randomized clinical trials have been published evaluating the efficacy of supplementation with progestogens in prevention of miscarriage.^5^ The groups of patients most studied in trials have been patients with prior recurrent miscarriage; those with symptoms of threatened abortion, such as first trimester vaginal bleeding; and those who underwent assisted reproductive technology (ART).^20^

### 2.1 Women with recurrent miscarriage

Recurrent miscarriage, or recurrent pregnancy loss, is defined as the loss of two or more pregnancies before 24 weeks.^21^ It has been hypothesized that a causative factor of first trimester abortion might be the inadequate secretion of progesterone during the luteal phase of the menstrual cycle and in the early weeks of gestation. A meta‐analysis of 10 randomized trials including 1586 women with recurrent miscarriage, failed to find high quality evidence of efficacy of progestogens in prevention of recurrent miscarriage.^5^ The main limitations of the meta‐analysis are related to the quality of the published studies. Trials used had small sample sizes, and were methodologically weak. Interventions used were different, including intramuscular natural progesterone, intramuscular 17‐hydroxyprogesterone caproate, oral medroxyprogesterone, oral quingestrone, oral dydrogesterone, or vaginal natural progesterone.^5^ Moreover, out of the 10 included trials, only three were published after 1990 and only two after 2010.

The only well‐powered randomized trial on the topic is the PROMISE trial.^22^ Coomarasamy et al. conducted a multicenter, double‐blind, placebo‐controlled randomized trial on the efficacy of 400 mg of micronized vaginal progesterone administered twice a day, from a time soon after receiving positive results on a urinary pregnancy test, and no later than 6 weeks of gestation, through 12 completed weeks of gestation, in patients with a history of unexplained recurrent miscarriages.^20^ In an intention‐to‐treat analysis, the rate of live births was 65.8% (262 of 398 women) in the progesterone group and 63.3% (271 of 428 women) in the placebo group. The authors found a 3% greater live birth rate with vaginal progesterone, but the results did not reach statistical significance. Notably, the trial showed a trend for greater benefit with an increasing number of previous miscarriages, generating the hypothesis that a subgroup effect existed with a biological gradient related to the increasing number of previous miscarriages.^20^

### 2.2 Women with threatened abortion

Vaginal bleeding is the most common symptom of threatened abortion. Heavy bleeding in the first trimester, particularly when accompanied by pain, is associated with a threefold increase in risk of miscarriage.^23^ Therefore, it has been proposed that vaginal bleeding can be used as a symptom to identify women at risk of abortion and, therefore, treated with progesterone in cases of luteal phase deficiency.

The PRISM trial^24^ is a large, randomized trial performed in 4153 women with first‐trimester vaginal bleeding, randomly assigned to receive either 400 mg of natural progesterone or matching placebo twice daily, from the time at which they presented with bleeding through to 16 weeks of gestation. The primary analysis of the PRISM trial found that the live birth rate was 75% in the progesterone group versus 72% in the placebo group, with no statistically significant differences. However, in the subgroup analysis of women with both vaginal bleeding and prior miscarriage, the authors found a trend of a benefit, which increases with an increasing the number of prior miscarriages.

### 2.3 Critical interpretation to PROMISE and PRISM trial

Both the PROMISE^22^ and PRISM^24^ trials enrolled heterogeneous populations, differing in clinical presentation, timing of randomization, and exposure to progesterone. The PROMISE^22^ trial included women with unexplained recurrent miscarriage and initiated treatment soon after a positive pregnancy test, while the PRISM^24^ trial enrolled women presenting with first trimester bleeding and treated them through 16 weeks. These differences in entry criteria, symptom status, and duration of therapy complicate direct comparison and might contribute to the minimal overall effect observed. In both trials, subgroup analyses suggested increasing benefit with a higher number of prior miscarriages and the presence of early bleeding, raising the possibility that progesterone might be effective only in well‐defined high‐risk patients. However, these findings could also reflect limited statistical power and multiple subgroup evaluations. The distinction between a true biological effect and statistical noise remains uncertain, reinforcing the need for carefully designed studies focused on women most likely to benefit.

## 3 CLINICAL IMPLICATIONS

Up to 70% of all first‐trimester abortions are related to genetic abnormalities.^25^ Trisomy, especially in patients with advanced maternal age, is the most common genetic cause, followed by polyploidy and monosomy. These abnormalities occur mostly on a random basis, excluding parental translocations, and therefore the risk of abortion in a subsequent pregnancy is not increased, most of the time. In contrast, euploid abortions are more frequently diagnosed with an increasing number of prior abortions. Specifically, it is the risk of euploid miscarriages that increases with an increasing number of previous miscarriages; meanwhile, the risk of miscarriage from sporadic aneuploidies remains broadly constant.

The biological plausibility of progesterone supplementation relies on the concept of luteal–placental shift, which occurs around 8 weeks of gestation, when the placenta rather than the corpus luteum becomes the primary source of progesterone. Beyond this point, supplementation might be less biologically justified, as circulating progesterone is already placenta derived. This raises concerns about the rationale for continuing treatment beyond 12 weeks of gestation, when endogenous production should be sufficient for the maintenance of pregnancy. Moreover, low progesterone levels in an early pregnancy might often reflect a non‐viable gestation rather than represent the primary cause of miscarriage.

There is no clear definition of luteal deficiency and no reliable tests to identify women at risk of luteal deficiency. The lack of a meaningful test for luteal deficiency raises the question of the target population for progesterone therapy. Therefore, there are two possible clinical approaches:

1. Universal supplementation with progestogens in all women in the first trimester of pregnancy. This approach can prevent miscarriage in women with luteal deficiency before they are identified as at higher risk and, therefore, before the diagnosis of explained recurrent miscarriage. However, this approach might lead to up to 95% of women treated unnecessarily because they would not have, in any case, a progesterone‐related abortion or would have a genetic‐related abortion,^26^ with social and economic consequences, including progesterone related side effects,^27^ such as headache, breast pain, vaginal discharge, tiredness, dizziness, constipation, increased hepatic transaminase,^28^ as well as increased risk of adverse effects in offspring related to synthetic progestins, such as 17‐OHPC,^29^ or dydrogesterone.^30^
2. Supplementation in women at risk of abortion. This approach would focus on the three groups of women at higher risk of abortion, including those with threatened abortion, recurrent miscarriage, or those who underwent ART.

## 4 ROUTES OF ADMINISTRATION

### 4.1 Vaginal versus intramuscular natural progesterone

Another issue is that if a clinician decides to offer natural progesterone therapy, they should consider the route of administration.

In the early days, natural progesterone was primarily offered by intramuscular injections. Intramuscular progesterone, while effective,^31^ can cause significant discomfort^32^ and require a trained healthcare provider for administration, potentially leading to issues with adherence.^32^ Additionally, the risk of injection site reactions and systemic side effects such as mood changes and headaches can deter patients from this route of administration.^33^ Therefore, alternative routes of administration have been studied, including oral, transdermal, vaginal, and subcutaneous.

Oral and transdermal progesterone have been shown not to be effective because the first is metabolized during the liver pass,^12^ while the transdermal route needs large amounts of progesterone.^13^ Hence, vaginal and subcutaneous administration appeared to be the only remaining alternatives to avoid painful injections, maintaining the same efficacy.^34^

Vaginal progesterone is the most studied progesterone for preventing preterm labor and preterm delivery,^31^ recommended by guidelines in women with short cervix^35^ and in those with prior preterm birth.^11^ Progesterone administered vaginally is associated with high uterine concentrations due to the direct vagina‐to‐uterus transport (first uterine pass effect),^36^ but low serum level, compared to intramuscular administration. Evidence from ART studies showed that women with low progesterone levels, at the time of embryo transfer, had lowered clinical pregnancy rates compared to higher progesterone levels.^37^ The most recent trials on vaginal progesterone did not account for progesterone levels and focused on late administration to prevent preterm labor, while early administration effects on spontaneous abortion are not well studied.^31^

Progesterone is crucial for maintaining pregnancy. It also has significant immunomodulatory properties that help regulate the maternal immune response during gestation.^19^ This immune adaptation is essential for the successful establishment and maintenance of pregnancy, particularly considering that the fetus, which is half derived from paternal genetic material, could potentially be recognized as a foreign entity by the mother's immune system. Understanding the immunomodulatory effects of progesterone, including how these effects might differ between vaginal and intramuscular routes of administration, is important for optimizing therapeutic strategies to prevent miscarriage and support pregnancy.^38^, ^39^, ^40^

The method of progesterone administration might influence its immunomodulatory effects.^33^, ^41^, ^42^, ^43^, ^44^, ^45^ Vaginal administration often results in higher local concentrations of the hormone in the reproductive tract.^36^ This localized delivery could provide a more direct effect on the immune cells present in the vaginal epithelium and the uterus, potentially leading to more pronounced effects on local immune modulation.^19^, ^37^ Additionally, the vaginal route might facilitate direct action on the endometrial tissue, creating an optimal immunological milieu for embryo implantation.^36^ In contrast, while intramuscular administration leads to systemic progesterone exposure and higher serum levels, it might result in different immune modulation dynamics. The levels of progesterone achieved in systemic circulation could potentially surpass local requirements, which might lead to a broader range of immunological effects, including those affecting peripheral immune responses.^41^

### 4.2 Subcutaneous progesterone

Subcutaneous progesterone administration has emerged as an alternative delivery route in the management of early pregnancy and the prevention of miscarriage, offering a novel approach. It is absorbed via the subcutaneous tissue into the bloodstream, resulting in a gradual and sustained release of the hormone.^46^, ^47^

The implications of subcutaneous progesterone are particularly relevant for women undergoing ART.^33^, ^43^, ^44^, ^45^ Women undergoing embryo transfer often require luteal phase support to ensure that the endometrium is adequately prepared for implantation. Subcutaneous formulations have been developed that are easy to administer, making them a practical option for patients. Randomized trials comparing subcutaneous progesterone to vaginal^43^ or intramuscular^33^ administration in women undergoing ART reported no significant differences in pregnancy rates or miscarriage rates, and better compliance,^44^ suggesting that subcutaneous delivery can be just as effective as traditional methods.

Clinical practice regarding progesterone supplementation in early pregnancy varies internationally. In some settings, progesterone, including oral formulations,^12^ is frequently recommended for threatened miscarriage despite the limited evidence for improvement in live birth outcomes. This divergence reflects differences in guideline development processes, access to care, and clinical priorities and highlights the ongoing debate between biologically plausible treatment strategies and the current limitations of randomized evidence.

## 5 COST‐EFFECTIVENESS

The feasibility and potential cost‐effectiveness of progesterone supplementation depend largely on the target population and on the route of administration.^48^ Universal treatment in early pregnancy would expose a high proportion of women to therapy despite the likelihood that many miscarriages are unrelated to progesterone deficiency or are genetically determined, raising concerns about unnecessary treatment burden, medication costs, and increased use of healthcare resources. Intramuscular progesterone requires administration by trained personnel and is associated with discomfort, injection site reactions, and systemic side effects, which might reduce adherence and increase the need for clinical support visits, further affecting feasibility.^49^ Vaginal formulations offer easier self‐administration but might still lead to local adverse effects and reduced compliance, whereas subcutaneous progesterone represents an emerging alternative with improved acceptability.^50^ Treating all pregnant women would therefore entail substantial logistical and practical challenges, including for medication supply, patient education, monitoring for adverse effects, and management of discontinuation in poorly tolerant individuals. In contrast, a selective approach focused on women at increased risk, such as those with early bleeding and prior miscarriage or recurrent pregnancy loss, might improve feasibility by limiting treatment to those most likely to benefit, potentially reducing unnecessary exposure and resource use.^48^ However, the absence of a reliable diagnostic tool to identify luteal deficiency remains a major barrier, as it prevents accurate selection and might reduce the efficiency of any supplementation strategy. Until better predictors of response and clearer identification of target populations are available, feasibility considerations support individualized treatment rather than universal administration, emphasizing shared decision‐making based on clinical history and patient preferences.

## 6 COUNSELING AND SHARED DECISION‐MAKING APPROACH

Given the limited overall benefit observed in randomized trials and the likelihood that many miscarriages are unrelated to progesterone deficiency,^51^ counseling should focus on individualized risk assessment and patient preferences. For women presenting with early bleeding and a history of prior miscarriage, clinicians might discuss that some evidence suggests a possible benefit in this specific subgroup, while emphasizing that the effect remains uncertain. In contrast, women without prior pregnancy loss or those undergoing their first pregnancy can be counseled that progesterone supplementation is unlikely to change outcomes in most cases, and that treatment might expose them to adverse effects and treatment burden without clear benefit. Counseling should include a discussion of administration routes, highlighting that intramuscular formulations require injections and might cause discomfort, whereas vaginal or subcutaneous options might offer greater convenience but can still lead to local side effects and adherence challenges. Shared decision‐making might therefore involve presenting the option of treatment, outlining potential advantages and limitations, and supporting women in choosing whether to initiate or continue progesterone supplementation based on their values, tolerance, and clinical history.

## 7 CONCLUSION

In summary, current evidence does not support universal progesterone supplementation in early pregnancy, and the absolute treatment effect observed in randomized trials appears small. Evidence published so far does not suggest significant benefit for progesterone therapy in women with early bleeding but no history of miscarriage. In contrast, progesterone therapy might be of benefit in those with early bleeding and prior miscarriage or in those with history of recurrent miscarriage. In any case, we recommend that available information should be shared and communicated to women at risk of first trimester abortion to enable shared decision‐making.

Future research is required to better understand the role of progesterone deficiency in early pregnancy loss and to develop a new test to identify patients at risk of luteal deficiency. Future studies should focus on the route of administration and also on a possible mixture of vaginal and injectable progesterone.

## AUTHOR CONTRIBUTIONS

Gabriele Saccone conceived the study concept, performed the literature review, and drafted the manuscript. Vincenzo Berghella contributed to the interpretation of evidence and critically revised the text for intellectual content. Moti Gulersen provided clinical and methodological input and participated in manuscript revision. Attilio Di Spiezio Sardo contributed to critical review of the manuscript and contextualization within current clinical practice. All authors approved the final version of the manuscript and agree to be accountable for all aspects of the work.

## CONFLICT OF INTEREST STATEMENT

The authors have no conflicts of interest to declare.

## Data Availability

Data sharing is not applicable to this article as no new data were created or analyzed in this study.
